# A single-centre, prospective, qualitative analysis of knowledge, attitudes and behaviour of sunbed use among patients attending a pigmented lesion clinic in a tertiary referral centre

**DOI:** 10.1093/skinhd/vzaf014

**Published:** 2025-05-06

**Authors:** Fei Ya Lai, Claire Quigley, Gregg Murray, Amanda Gordon, Ji Fung Yong, Helena Yoo, Claudine Howard-James, Kelly Impey, Carmel Blake, Anne-Marie Tobin

**Affiliations:** Dermatology Department, Tallaght University Hospital, Dublin, Ireland; Dermatology Department, Tallaght University Hospital, Dublin, Ireland; Dermatology Department, Tallaght University Hospital, Dublin, Ireland; Dermatology Department, Tallaght University Hospital, Dublin, Ireland; Dermatology Department, Tallaght University Hospital, Dublin, Ireland; Dermatology Department, Tallaght University Hospital, Dublin, Ireland; Dermatology Department, Tallaght University Hospital, Dublin, Ireland; Dermatology Department, Tallaght University Hospital, Dublin, Ireland; Dermatology Department, Tallaght University Hospital, Dublin, Ireland; Dermatology Department, Tallaght University Hospital, Dublin, Ireland

## Abstract

**Background:**

Indoor tanning through sunbeds is linked to a heightened risk of skin cancers, particularly cutaneous squamous cell carcinoma and basal cell carcinoma, with significant increases in risk for users aged < 35 years. Despite regulations established by the Public Health (Sunbeds) Act 2014 in Ireland, sunbed use persists, primarily for cosmetic reasons.

**Objectives:**

To analyse the characteristics, attitudes and behaviours of sunbed users attending an Irish dermatology outpatient clinic.

**Methods:**

We undertook a prospective qualitative analysis of 104 consecutive patients attending a pigmented lesion clinic in a tertiary referral dermatology department in Ireland. This was done using a self-reported anonymous survey where respondents answered questions relating to their own demographic data, frequency of sunbed use, motivation for sunbed use and use of unregulated tan-enhancing agents (namely Melanotan I and II).

**Results:**

The results showed that patient demographics were consistent with previous studies on sunbeds use, namely younger female patients living in urban areas. Many sunbed premises did not comply with safety regulations; over half lacked protective goggles, and nearly half received no health risk information. The reasons for sunbed use included improving appearance and confidence, with a significant number using tan-enhancing agents. Surprisingly, increased awareness of health risks did not correlate with reduced usage; many users continued tanning practices despite concerns about its adverse effects. Users of tan-enhancing agents also used sunbeds more frequently compared with nonusers.

**Conclusions:**

This study suggests a potential psychopathological aspect of tanning behaviours similar to addictive disorders like smoking and alcohol. Patients may benefit from psychological and behavioural interventions such as cognitive behavioural therapy to address their compulsive behaviour. Furthermore, there was a concerning lack of compliance with regulations in tanning salons, highlighting a public health issue. The rising use of unregulated tanning agents, especially among younger people, poses additional risks, including blood-borne infections. This study underscores the need for targeted educational interventions among younger age groups and stricter enforcement of regulations to mitigate health risks associated with indoor tanning. Understanding the complex motivations behind sunbed use is crucial for developing effective strategies to reduce its prevalence and promote safer alternatives.

## Teaser

Awareness or concerns on adverse effects of sunbed does not correlate with lower usage.

The addictive aspect of sunbed use for achieving a desired cosmetic appearance suggests a compulsive behaviour which needs to be studied in more detail. Lack of strict regulation on sunbed premises and unregulated tan-enhancing agents are important public health concerns, contributing to increasing incidence of skin cancer including melanoma and other adverse health outcomes.

**What is already known about this topic?**

- Regular sunbed users are usually younger female individuals, with lower knowledge of risks of sunbed use, motivated by increased confidence through tanned skin.

**What does this study add?**

- Awareness of adverse outcomes of sunbeds does not correlate with lower usage.
- The addictive aspect to sunbed use has not been explored, and patients may benefit from psychological and behavioural interventions (e.g. cognitive behavioural therapy).

Indoor tanning has long been associated with increased risk of developing skin cancer. Outside of developing countries, sunbeds are the most frequent source of ultraviolet radiation. Furthermore, sunbed use is associated with other adverse health outcomes such as burns, ocular diseases, immunosuppression and premature skin ageing.^1^ According to the Irish Cancer Society, one sunbed session increases the risk of developing cutaneous squamous cell carcinoma by 67% and basal cell carcinoma by 29%. This risk of melanoma increases to almost 60% if a person uses sunbeds before the age of 35 years.^2^

In Ireland, the Public Health (Sunbeds) Act 2014 was introduced to protect sunbed users and promote informed decisions. Current regulations include prohibition of the use, hire or selling of sunbeds to persons < 18 years of age; the use of protective clothing, health warning signage, prohibitions of health claims by sunbed premises, as well as the prohibition of certain marketing practices.^2,3^

However, despite sunbed regulation and efforts by government bodies and the health service to promote awareness of the hazards associated with indoor tanning, sunbed use is still common among the Irish population to achieve a desired cosmetic outcome, despite availability of other safe options (e.g. tanning creams). A survey carried out in 2017 by the Irish Cancer Society showed that 150 000 Irish people have used a sunbed within the last year. Understanding the determinants of sunbed use is vital to plan educational interventions, behavioural strategies and regulatory measures that target specific subgroups with different risk profiles.

The aim of our study was to identify the characteristics of sunbed users, assess their attitudes and behaviours toward indoor tanning and their awareness of its health risks, and factors that promote its use among patients attending an Irish dermatology outpatient clinic.

## Patients and methods

We undertook a prospective qualitative analysis of 104 consecutive patients attending a pigmented lesion clinic in a tertiary referral dermatology department in Ireland. This was done using a self-reported anonymous survey where respondents answered questions relating to their own demographic data, frequency of sunbed use, motivation for sunbed use and use of unregulated tan-enhancing agents (namely Melanotan I and II). The survey responses were collected and then analysed using descriptive statistics and linear correlation analysis.

## Results

### Patient demographics

Twenty-eight male and 76 female individuals participated in the study (age range 17–76 years). Of the 104 patients surveyed, 86 (82.7%) reported previous sunbed use. Female participants (*n* = 69/76, 90.8%) were more likely to use sunbeds than male participants (*n* = 19/28, 67.9%). The majority of respondents had Fitzpatrick skin type III (*n* = 61/104, 58.7%), followed by skin type II (*n* = 22/104, 21.2%), skin type I (*n* = 11/104, 10.6%), skin type IV (*n* = 9/104, 8.7%) and skin type VI (*n* = 1/104, 1.0%). The majority (*n* = 59/86, 68.6%) of sunbed users were younger than 22 years old. Twenty-two patients (*n* = 22/86 25.6%) were under the legal age of 18 years. The earliest reported age at first use was 13 years.

### Adherence to regulations

Most tanning beds were available in urban areas (79.8%), with 66 of 104 patients (63.5%) reporting having 1–5 tanning salons around them. Approximately half of sunbed users (*n* = 49/86, 57.0%) accessed sunbeds in tanning salons, a further 25 (29.1%) had access to a sunbed at home or in the house of a relative or friend, 7 patients (8.1%) accessed a sunbed through their local beauty salons and 1 patient (1.2%) used a sunbed in a gym (16 patients did not specify where they used sunbeds). Thirty-one (29.8%) respondents reported not wearing protective goggles, and 24 of 104 (23.1%) patients did not have staff supervision during their tanning sessions. Fifty of 104 patients (48.1%) were not given any safety or health risk information prior to using sunbeds (Table 1).

**Table 1:** Compliance of sunbed premises to regulations

Response	Protective goggles	Staff supervision	Information on safety and health risk
Yes	43 (41.3)	58 (55.8)	27 (26.0)
No	31 (29.8)	24 (23)	50 (48.1)
Blank/cannot remember	30 (28.8)	22 (21.2)	27 (26.0)

Data are presented as *n* (%).

### Tanning behaviours and attitudes

**Figure 1:**
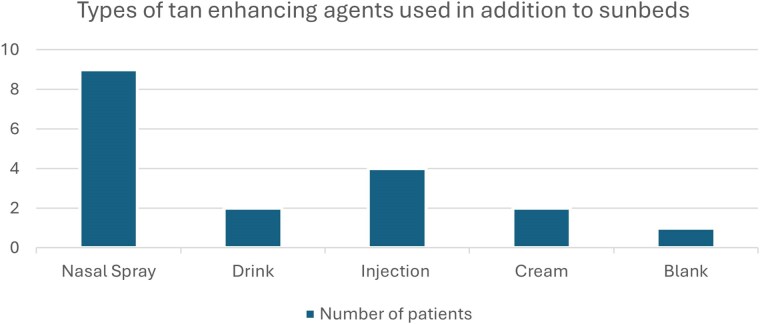
Types of tan-enhancing agents used.

Sixty-one per cent (*n* = 63/104) of respondents used sunbeds at least once a week. More than half of respondents (*n* = 57/104, 54.8%) use sunbeds for 5–10 min per session. Twenty-six of 104 patients (25.0%) used sunbeds for 10 min or longer. Eighteen of 104 patients (17.3%) reported using adjunct agents to augment their tan such as nasal sprays, injections and tanning drinks (Figure 1). Users of tan enhancers also used sunbeds more frequently than nonusers (Table 2).

**Table 2:** Comparison of frequency of sunbed use in patients who are users and nonusers of tan enhancers

Frequency of sunbed use	Number of patients using tan enhancers (*n* = 18)	Number of patients who do not use tan enhancers (*n* = 86)
2–3 times weekly	11 (61.1)	24 (27.9)
Once weekly	4 (22.2)	20 (23.3)
Every 2 weeks	0	3 (3.5)
Every month	2 (11.1)	1 (1.2)
Every 2–6 months	0	4 (4.7)
Once a year	1 (5.6)	4 (4.7)
Before special occasions/holidays	0	10 (11.6)
No answer	0	20 (23.3)

Data are presented as *n* (%).

**Figure 2:**
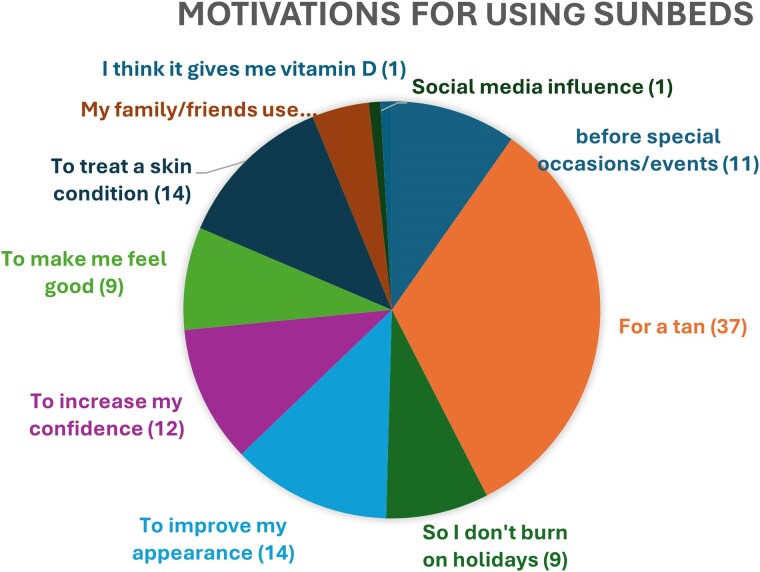
Motivations for using sunbeds among patients surveyed.

The majority of patients (*n* = 37/104, 35.5%) reported a goal of achieving a tan to improve their overall confidence and appearance as a motivational factor. One respondent reported the goal of increasing their vitamin D level as a reason for sunbed use. Social media and the influence of family and friends were found to be low-risk motivating factors (<1% and 4.8%, respectively) (Figure 2). The majority of these patients (*n* = 63/104, 60.6%) were worried about the risk and side-effects of using sunbeds. Among those who expressed concern about side-effects of sunbeds (*n* = 63), 73.0% (*n* = 46/63) were using sunbeds at least once a week; 24/63 patients (38.1%) had used sunbeds within the last 6 months (Tables 3, 4).

**Table 3:** Comparison of interval sunbed use among patients concerned about the side-effects of sunbeds

Interval of sunbed use	Individuals worried about sunbed use (*n* = 63)
Last week	11
Last month	8
1–6 months ago	5
6–12 months ago	2
More than a year ago	37

**Table 4:** Comparison of frequency of sunbed use among patients concerned about the side-effects of sunbeds

Frequency of sunbed use	Individuals worried about sunbed use (*n* = 63)
2–3 times a week	28
Once a week	18
Every 2 weeks	2
Every month	2
Every 2–6 months	2
Once a year	4
Before special occasions	4
Not specified	3

Categorical responses were further numerically coded, and Pearson’s correlation coefficient was used to assess relationships between key variables, including frequency of use, skin burning and risk awareness. The correlation between worrying about side-effects and how often someone uses sunbeds was low (*r* = 0.07), suggesting a weak or no relationship. There was a low negative correlation (*r* = −0.15) between worrying about side-effects and whether they were informed about health risks, indicating a slight tendency for those informed about risks to worry less. ‘Did your skin ever burn?’ had a small positive correlation (*r* = 0.25, *P* < 0.05) with how often people use sunbeds, meaning frequent users are slightly more likely to have experienced burning. A positive correlation was identified between the frequency of sunbed use and the occurrence of skin burns (*r* = 0.25, *P* < 0.005). The association was statistically significant (*P* < 0.005), suggesting that frequent sunbed users are at an increased risk of experiencing burns.

## Discussion

Several factors influence sunbed use. Previous studies have highlighted increased sunbed use among young female individuals, those from higher socioeconomic backgrounds and those with reduced knowledge on the health risk associated with regular sunbed use. In addition to this, these studies have also identified improved confidence and positive emotions associated with sunbed use as motivational drivers.^4–7^

The principal finding of our study was that increased knowledge and awareness of the risks associated with sunbeds do not correlate with reduced frequency of sunbed use among sunbed users. Patients who were aware of the risks associated with indoor tanning continued using sunbeds despite knowledge of its potential for adverse health outcomes. The reason behind this conflicting behaviour pattern is not fully understood, but we postulate that an emotional and physical addiction to sunbeds may play a role. One study by Aubert *et al.*^8^ highlighted an increased efflux of dopamine release (which plays an important role in the brain reward pathway) among frequent sunbed users compared with infrequent users, suggesting a cutaneous–­neural connection driving continued sunbeds use. This neural pathway can also be seen in other addictive disorders, suggesting a compulsive behaviour similar to smoking or alcohol use. Furthermore, a study by Petit *et al.* showed excessive indoor tanners met the substance abuse and dependence criteria (Diagnostic and Statistical Manual of Mental Disorders, Fourth Edition, Text Revision), highlighting the psychopathology aspect of tanning dependence of some users.^9^

Our study also highlighted a lack of compliance with sunbed regulations in Ireland. Respondents reported that some premises did not provide safety goggles (which increases the risk of developing ocular melanoma), staff supervision or health and safety information, and there were no age limitations in providing indoor tanning services, despite the legal requirements.^10^ Countries such as Brazil, Australia and Iran have banned the commercial use of tanning salons; however, it is still legal for those aged 18 years and above in the USA and the majority of European countries.^7,11^ It was outside the scope of our study to identify whether or not the premises our patients were attending were registered with the national sunbed office; however, attendance and use of sunbeds at unregistered and unregulated salons is certainly a public health concern.

The use of tanning adjuncts such as nasal spray, injections, drinks, Melanotan I and II (which contains a synthetic version of melanocyte-stimulating hormone when exposed to ultraviolet radiation) are also becoming more popular among sunbed users, especially among the younger population, who are more likely to be influenced by their social environments.^11,12^ There is not only an increased risk of developing skin cancers, but also other potential health risks such as blood-borne infections via needle sharing.

This study highlights the need for more stringent legislative measures in regulating premises providing indoor tanning services and selling of other tanning agents. Regulating the availability of sunbed premises within each local area, either outside or within a person’s home, may help limit access and thus frequency of usage. Educational campaigns need to target teenagers and young adults given the prevalence of sunbed use among these age groups, for example by carrying out awareness campaigns in schools or universities. Furthermore, there needs to be more regulation on the selling of potentially harmful tanning adjuncts especially to vulnerable groups. However, more importantly we need to look at potential psychosocial and behavioural interventions such as cognitive behavioural therapy to break the addictive cycle of sunbeds use and people’s perception of its physical and emotional benefits.

## Data Availability

The data underlying this article are available in the article.
